# Low carbon transportation in Thailand: CO_2_ mitigation strategy in 2050

**DOI:** 10.1186/s40064-015-1388-6

**Published:** 2015-10-16

**Authors:** Puttipong Chunark, Panida Thepkhun, Kamphol Promjiraprawat, Pornphimol Winyuchakrit, Bundit Limmeechokchai

**Affiliations:** Sirindhorn International Institute of Technology, Thammasat University, Pathumthani, Thailand

## Abstract

Nationally Appropriate Mitigation Actions (NAMAs) involve the collaboration on reduction of greenhouse gas (GHG) emissions in developing countries with suitable countermeasures relevant to the state of technological and economic conditions prevalent in the country. This study proposes appropriate GHG countermeasures in Thai transport NAMAs, which are based on the implementation of transport demand management, modal shift, fuel switching, and advanced technologies in the timeframe between 2005 and 2050. Furthermore, this study considers the impacts of CO_2_ mitigation through the proposed countermeasures on energy security and GHG emissions. Results of analyses on low carbon transportation are also useful to other developing countries. Finally, the concept of marginal abatement cost is employed to investigate cost-effective mitigation countermeasures.

## Background

Since the world oil crisis in the 1970s, researchers and policy makers have started understanding and investigation on energy savings and management in the energy systems, especially power generation, industry, and transport sectors. In addition, the climate change problem has provided greater motivation to improve scientific discourse. Hence, not only investigation of greenhouse gas (GHG) mitigation potential but also study of mitigation schemes is an important issue, which has been extensively discussed in the field of energy planning and environmental management.

The United Nations Framework Convention on Climate Change (UNFCCC), which was established in 1992, is an international environmental treaty to set binding obligations on industrialized countries to reduce GHG emissions as well as to achieve the stabilization of GHG concentration in the atmosphere (UNFCCC 2005). The Kyoto Protocol (KP) has been adopted to reduce both direct and indirect GHG emissions (UNFCCC 2008a). The GHG data reported by UNFCCC contain estimates for the direct greenhouse gases such as carbon dioxide (CO_2_), methane (CH_4_), nitrous oxide (N_2_O), perfluorocarbons (PFCs), hydrofluorocarbons (HFCs), and sulphur hexafluoride (SF_6_) as well as for the indirect greenhouse gases such as sulfur dioxide (SO_2_), nitrogen oxides (NO_x_), carbon monoxide (CO), and non-methane volatile organic compounds (NMVOC) (UNFCCC 2013b). In the context of GHG emissions target as part of the KP, Annex I countries have agreed to legally binding limits on their emissions in two commitment periods. In order to accomplish the emission reduction targets of the first commitment period of the KP, commencing in 2008 and ending in 2012, each Annex I party has a binding commitment to reduce or limit GHG emissions and to develop mechanisms to facilitate observance of this commitment (UNFCCC 2012). The first commitment period ended with the result that the United States signed but did not ratify the protocol, and Canada withdrew KP in 2011. In the second commitment period (2013–2020), the protocol was modified in 2012; however, this modification has not entered into the legal force but remains in the negotiation process of some Annex I countries which refuse to accept the legal force procedure (UNFCCC 2012).

In order to achieve the emissions target to limit the increase in global average temperature to a maximum of 2 °C above pre-industrialized levels, the 16th Conference of the Parties (COP16) or “Cancun agreement” requires that the Annex I parties reduce emissions in the range of 25–40 % below 1990 levels by 2020 towards building a low-carbon society. A low-carbon society (LCS) or low-fossil-fuel economy is a concept that refers to an economy which has a minimal output of GHG emissions into the biosphere. LCS also stands for the low-carbonization in all aspects of a society including economy, culture and life (Yuan et al. 2011), but specifically refers to the greenhouse gas carbon dioxide (Winyuchakrit et al. 2011a). However, in 2010, the Cancun agreement recognized that the developing countries should contribution to the mitigation endeavor in the context of “sustainable development” to meet their emission target. The LCS concept has been adopted by developed countries, e.g., establishing a mandatory target of renewable energy consumption in the European Union (Carvalho et al. 2011). The target of GHG mitigation has been committed by Japan and the United States in Japan-U.S. Summit Meeting (2009), etc. In developing counties, there are a variety of geographic features, cultures, incomes and life styles, and developing countries are also concerned about sustainable development through the LCS mechanism as shown in a number of relevant studies. For instance, the proposed low mitigation scenarios (2.6 W/m^2^) are employed by India and China to achieve LCS (Calvin et al. 2012), the benefits of low carbon development of Nepal were analyzed through the MARKAL model (Sherstha and Shakya 2012), etc. Presently Thailand is a developing country and categorized as non-Annex I country that has no commitment to any quantitative objectives under the KP but realizes that achieving those objectives is the common problem of all countries. Thus, Thailand has designed a low carbon society within the context of sustainable development using the approach of Nationally Appropriate Mitigation Actions (NAMAs), which has been intensively studied in order to prepare and propose achievable measures for sustainable development. Sritong et al. (2011) presented the potential of renewable power generation in Thailand for CO_2_ mitigation under the framework of NAMAs. Additionally, Winyuchakrit et al. (2011b) gathered the information concerning GHG mitigation strategies and analyzed the opportunities for reducing GHG emissions in Thailand in 2020.

NAMAs, which involve the collaboration on reduction of GHG emissions in developing countries with the suitable countermeasures regarding national circumstances, were first mentioned in the 13th session of the Conference of the Parties (COP13) or “Bali Action Plan” as part of the “Bali Roadmap” under the decision of UNFCCC (2008b). NAMAs can be performed in two categories depending on sources of funding, which are “domestically supported mitigation actions (DSMAs)” and “internationally supported mitigation actions (ISMAs)”. DSMAs are the unilateral NAMAs whose finance and implementation are supported by the government of the country. The countermeasures of DSMAs generally are of low investment costs and can be implemented by domestic efforts, such as energy management and energy efficiency improvement. Furthermore, DSMAs will be domestically measured, reported, and verified (MRV). On the other hand, ISMAs are the mitigation actions seeking international support in order to facilitate matching of finance, advanced technology, and capacity building. ISMAs mostly are the actions that require high investment cost and advanced technology.

Measuring, Reporting and Verification of NAMAs is the critical component framework for tracking emission mitigation actions and financial support which being implemented. Moreover, MRV framework can illustrate the transparency and reliability between financial supporters provided by developed countries and implemented actions by developing countries, to confirm that the financial support and knowledge have been effectively transferred, applied and executed GHG mitigation actions to keep the increase in average global mean temperature under 2 °C (Bakker and Huizenga 2010a; CCAP 2011; CCAP 2012; UNFCCC 2013c). MRV can be important either sharing the best practices and financial support for implementing actions or focusing on the different perspectives of mitigation actions (Bakker et al. 2010b; Neuhoff 2009). UNFCCC has provided good practices to be taken into account for designing a measurement methodology including accuracy, completeness, conservativeness, consistency, comparability and transparency (UNFCCC 2013c). Reporting is also an import part for MRV which can be reported into two different elements namely; reporting regularly and clearly illustrating peers in order to allow an evaluation and feedback (UNFCCC 2013c). Verification is an essential mechanism for checking the accuracy and the reliability of information and the procedure for generating such information in order to facilitate the transparency (UNFCCC 2013c).

Thailand is a developing country which has lower per capita emissions and lower emissions per gross domestic product (GDP) compared to developed countries. Thailand is also facing challenges in energy-environment-economy development in the context of limited energy resources availability and global climate change. The energy situation which has been reported by the Ministry of Energy (MOE) states that the total final energy consumption has increased annually by about 1.76 % during 2007–2011 (DEDE 2010). By economic categories, the transport sector in Thailand was the sector with the highest energy consumption of 25,469 kt of oil equivalent (ktoe) and accounted for 36.10 % of the total final energy consumption in 2011 (DEDE 2011). Petroleum fuels and gases, i.e., compressed natural gas (CNG) and liquefied petroleum gas (LPG), are the main energy sources consumed in this sector. In the past, fuels have been mainly consumed whereas gases consumption was a minor share. Due to the increase in world oil price, gases consumption in the transport sector had significantly increased from 3.70 % in 2007 to 12.20 % in 2011. Fuels have been the main energy resource in the sector but their structure has been significantly changed in recent times. Since benefits of biofuel consumption include savings of fossil fuels as well as GHG emissions mitigation (Malca and Freire 2009), policies on biofuel consumptions are promoted, such as the 10-year alternative energy development plan (AEDP) during 2012–2021, in which ethanol and biodiesel have been blended in petroleum-based gasoline and diesel, respectively. Blended fuels have become the priority share, especially ethanol mixed in gasoline or so-called gasohol. Although biofuel has been an important alternative source, Thailand imports energy at continuously increasing rates. In 2011, energy import value was 41.2 million USD and accounted for 29.15 %, increasing from the previous year. The volume of crude oil and petroleum products accounted for 83.00 % of all imported energy and their refinery products are mostly consumed in the transport sector (DEDE 2011). For this reason, the AEDP during 2012–2021 has been announced in order to increase the target of energy consumption reduction and GHG mitigation (DEDE 2012).

Therefore, the objectives of this study are to investigate the feasibility of proposed countermeasures and to develop an appropriated GHG mitigation plan for the passenger and freight transport sectors under NAMAs perspectives through the AIM/Enduse modeling. Moreover, this study focuses on direct emissions generated at the exhaust pipe of vehicle types and at stacks of power plants for the GHG mitigation assessment. The proposed mitigation countermeasures are based on (1) transport demand management, (2) modal shift, (3) fuel switching, and (4) implementation of advanced technologies. Additionally, to assure the path of sustainable development via these countermeasures, energy security and other air pollutions have been considered in many recent studies (Shakya and Shrestha 2011; Shrestha and Pradhan 2010; Sujeetha and Limmeechokchai 2013), thus they are presented to analyze further GHG emissions. Finally, marginal abatement cost (MAC) is employed to indicate the investment potential of mitigation actions that would be categorized into DSMAs and ISMAs of NAMAs.

## Methodology

The policy analysis presented in this study relies on the principle of the conventional transport planning, which is demand oriented. The objective of this planning is to determine the optimal implementation of modal shift, fuel switching, and advanced technologies that meet both passenger and freight transport demands while satisfying all given constraints.

**Fig 1:**
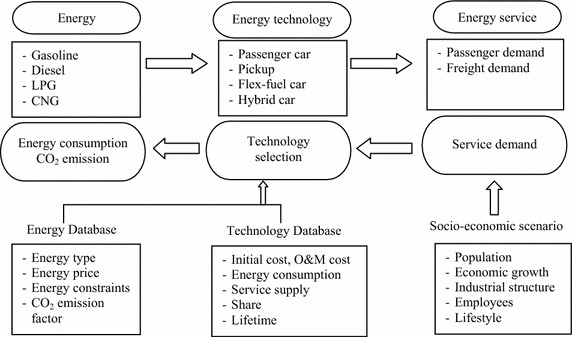
Schematic of AIM/Enduse model for Thailand’s transport sector

The Asia–Pacific Integrated Assessment Model/Enduse (AIM/Enduse) developed by the National Institute for Environmental Studies (NIES), is employed in this study. The model relies on a framework of linear programming to structure the bottom-up energy system which begins with how much primary energy source is converted into secondary energy to be required by an end-use service. GHG emission is also estimated by energy consumption of a particular technology. Based on the concept of a dynamic recursive model to solve the problem for multiple years, the total system cost of technology selection is minimized year by year subject to various related constraints. The total cost comprises capital cost of purchasing equipment in specific service types, operation and maintenance requirement, and energy cost. In addition, the model provides a spreadsheet interface to analyze several constraints such as satisfying service demand growth, preparing energy resource, reducing pollutant emission, and ensuring equipment stock (Akashi et al. 2011; Shrestha et al. 2007; NIES 2012; Pandey 2002). The schematic of the AIM/Enduse model for Thailand’s transport sector is illustrated in Fig. 1.

**Fig. 2:**
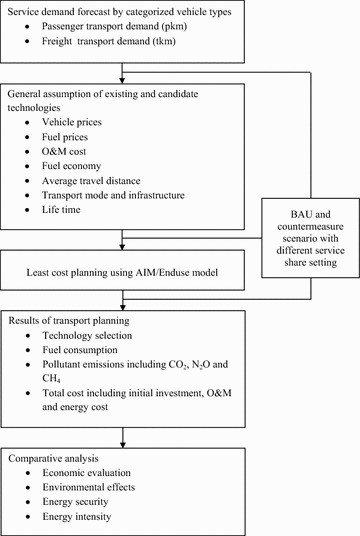
General framework of the study

In this study, the passenger and freight transport sectors have been modeled to estimate CO_2_ mitigation potential through the AIM/Enduse. As mentioned in the previous section, the business as usual (BAU) and the Low Carbon Transport (LCT) scenarios are considered and compared to analyze different perspectives including CO_2_ mitigation potential and energy security as well as economic evaluation. The general framework of this study is portrayed in Fig. 2. The methodology starts with estimation of service demand which is satisfied by employing particular technologies in particular sub-sectors. Both scenarios are represented as passenger and freight transport demands in terms of passenger–kilometer (pkm) and ton–kilometer (tkm), respectively.

Since fuel consumption of transport vehicles is definitely correlated to various pollutant emissions, technology selection would play an important role for reducing fuel requirement and pollutant emissions. However, inexpensive technologies are always required for the modern lifestyles in developing countries. The least cost model is able to solve these problems but several constraints need to be taken into account, especially a limitation of pollutant emissions. Economics and technical information of all technologies are observed to define reliable parameters for optimization. Apart from the fixed costs of particular technology, including installation cost, operation and maintenance cost, corresponding energy costs are also considered for setting the coefficient in the objective function. Energy cost of a particular technology is related to its own energy efficiency. In some technologies, energy cost might dominate the initial investment; when a technology lifetime is long enough, that means low-energy-cost technology can provide more cost saving than a technology with low investment cost. Selected technologies in the AIM/Enduse model are linked to corresponding energy consumption which satisfies the service demand in specific sub-sectors and also correlates to the pollutant emissions. The total cost of technology is also required to determine the marginal abatement cost.

### AIM/Enduse modeling

**Fig. 3:**
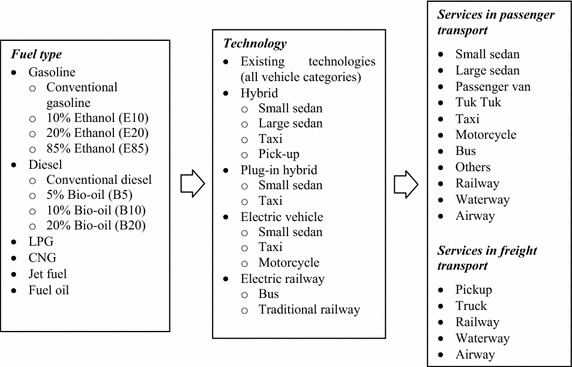
Structure of passenger and freight transport sectors in the model

In the AIM/Enduse model structure, three integral components, “Energy”, “Device”, and “Service”, need to be defined and linked in order to simulate the energy system. Technology is a linkage between energy and service. Generally, a technology consumes energy to produce a desirable output which satisfies service demand. Figure 3 shows the structure of the AIM/Enduse model which is applied in the passenger and freight transport sectors. The passenger transport of Thailand has been divided into 11 vehicle types namely; small-sedans, large-sedans, passenger vans, tuk-tuks (tri-wheeled motorcycles), taxis, motorcycles, buses, railways, electric trains, airways, and other miscellaneous vehicles. On the other hand, the freight transport in Thailand contains 5 vehicle types including pick-ups, trucks, railways, airways, and waterways, respectively.

Various mitigation actions are able to reduce the fuel consumption and the pollutant emissions. Introduction of more effective and efficient technologies has been investigated to determine their mitigation potential and other impacts such as economic and other air pollutions. Development in vehicle technology has a long and successful history. Up to now, there exist various alternatives to be selected in both passenger and freight transports. Hybrid vehicles were first launched in 2010 and will be an attractive candidate technology for sedans, taxis, passenger vans, and pick-ups for providing energy efficiency improvement and CO_2_ emission reduction. Electric vehicle is an option to reduce imported oil and fossil fuel dependency. However, it is not easy to investigate CO_2_ emission reduction because it depends on the technology used to generate electricity.

Additionally, fuel switching plays an important role in reducing undesirable dependency on fuel imports and high carbon content energy. In the case of Thailand’s transportation, petroleum products blended with biofuel such as ethanol has been given serious attention to investigate the feasibility of blended products (Pongthanaisawan and Sorapipatana 2011).

### Transport demand forecast

**Fig. 4:**
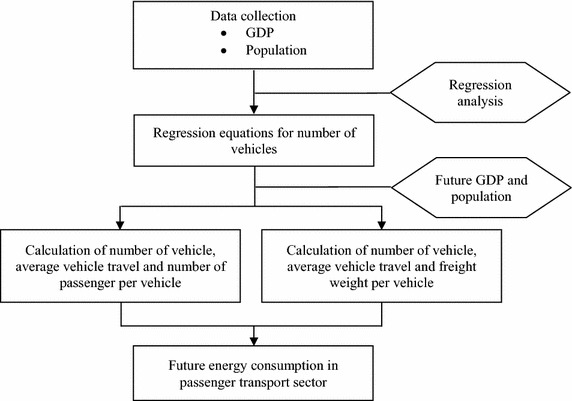
Flow diagram of energy demand projection in the passenger and freight transport sector

The energy demand projection has been carried out using regression analysis in terms of number of vehicles by type, and the steps of the projection are shown in Fig. 4. The population and GDP from 1990 to 2010 have been used as independent variables. The relationships of the energy consumed by vehicle types from 1990 to 2010 are obtained as regression equations related to those variables. Thailand’s total GDP was accounted for 170,202 million USD in 2005 and transport’s value added was accounted for 14,328 million USD in 2005 (2002 as the reference year) (NESDB 2012). While the exchange rate was obtained from Bank of Thailand (BOT 2013). The total GDP has been projected with the help of growth rates given in the Power Development Plan; PDP 2010 (EPPO 2012) referring to the trajectory by the Office of National Economic and Social Development Board (NESDB).

The population growth rate has been estimated from the historical data at 0.51 % per annum during 2011–2050. Then the GDP and population are projected to determine energy consumption for each vehicle type. In order to obtain the physical unit of energy demand for passenger transport as pkm, average vehicle travel and number of passengers per vehicle are required for each passenger vehicle type. In addition, energy demand for passenger transport as tkm can be obtained by multiplication of average vehicle travel and freight weight per vehicle.

Regression equations for estimation of the number of vehicles, in terms of their coefficients and coefficient of correlations (R^2^), are given in Table 1. This study formulates the multiple variable linear regression method to estimate energy consumption.

**Table 1:** Coefficients of estimated number of vehicles

Sector	A	B	c	R^2^
Small sedan	56.636	0.75026	−28,250,925	0.9832
Large sedan	34.376	0.46688	−17,147,011	0.9832
Passenger van	1.930	0.28337	−94,588	0.9996
Tuk tuk	0.212	0.00698	172,208	0.9311
Taxi	1.196	−0.00241	−415,409	0.9479
Motorcycle	−36.701	0.00770	−101,166,717	0.9794
Bus	0.638	1.86927	−122,061	0.9987
Pick-up	73.948	0.44702	−25,326,736	0.9835
Truck	5.269	0.05679	−2,928,072	0.9866
Others	8.584	0.00383	44,329	0.6551
Railway	1.305	0.00064	−58,867	0.9970
Electric railway	−3708.478	1.00121	−1,705,321,643	0.9360
Waterway	0.102	0.00201	−118,723	0.9669
Airway	0.009	0.00002	−1061	0.9733

The respective equation for multiple variable regressions is:

1 $${\text{A number of vehicle }} = \, A \times \;({\text{GDP}}) \, + \, B \times \;({\text{Population}}) \, + \, c$$

where *A* is the coefficient of the independent variable of value added for the respective sub-sectors in USD million (at constant price, year 2002).

*B* is the coefficient of the independent variable of population of Thailand.

*c* is the constant.

The service demand of the passenger transport sector, obtained by using the regression analysis method espoused above, is given in Table 2.

**Table 2:** Service demand in Thailand transport sector

Sub-sectors	Service demand in the passenger transport sector (Million-pkm)			
	2005	2020	2030	2050
Sedan	39,227	109,352	165,024	285,789
Small sedan	27,210	75,853	114,471	198,241
Large sedan	35,636	42,633	48,070	59,896
Passenger van	904	629	457	89
Tuk tuk	4540	7522	9979	15,313
Taxi	99,337	156,466	196,799	283,740
Motorcycle	138,310	172,991	199,286	256,388
Bus	1290	3760	5162	8229
Others	11,582	9239	7565	4753
Railway	184	260	308	409
Airway	3625	8189	12,159	20,789
Total	361,844	477,542	759,281	1,133,635

Sub-sectors	Service demand in the freight transport sector (Million-tkm)			
	2005	2020	2030	2050
Pick-up	64,267	126,840	175,618	281,531
Truck	105,702	157,078	199,145	290,326
Railway	2958	2360	1932	1214
Waterway	1,628,732	3,551,676	5,110,529	8,484,102
Airway	24,972	56,418	83,768	143,224
Total	1,826,631	3,894,371	5,570,992	9,200,397

### Energy security and other air pollutions

Energy security indicators represent the ability of an economy to guarantee the availability of energy resource supply in a sustainable and timely manner (APERC 2007). Energy security indicators for this study comprise energy diversity (ED), energy intensity (EI), energy saving per capita (ES) and carbon intensity (CI). The EI represents the energy use in the transport sector per GDP and ES represents differences in energy demand which occur in the BAU and LCT scenario per population. In the environmental aspect, the CI indicator represents the proportion of CO_2_ emissions per GDP. These indicators have been given on a scale of 0–100, where 0 indicates minimum security whereas 100 indicates maximum security, and they are represented for passenger and freight vehicle contexts in the base year (2005), NAMAs target year (2020), medium-term year (2030) and long-term year (2050).

The energy diversity (ED) index considers both the significance of diversification in terms of abundance and equitability of energy sources. The ED indicator in passenger and freight vehicles demand has given by adopting the Shannon Index, *H*′, which is a diversity index used to measure biodiversity (APERC 2007) as in the equation:

2 $$H^{\prime} = - \sum\limits_{i = 1}^{R} {p_{i} \ln p_{i} }$$

where *p*_*i*_ is the proportion of characters belonging to the *i*th of energy supply.

The ED index is calculated by the following equation:

3 $$ED = \frac{{H^{\prime}}}{\ln T}$$

where *T* is number of energy sources which are utilised.

In addition, the increasing of energy demand and CO_2_ emissions against population growth rate are analyzed through the proportion of energy per capita (ECap) and CO_2_ emission per capita (CECap). The ECap and CECap indicators are represented for passenger and freight vehicles during 2005–2050.

The greater number of ED is, the better energy diversity has been consumed in the transport sector. The EI is used for considering the penetration of energy efficient technologies compared to GDP. As countermeasure scenario has been presented, there are plenty of alternative fuels used in this scenario by efficient technologies. The lesser the number is, the better energy efficient technologies are introduced. Moreover, the efficient technologies in this study have introduced the vehicle used gasoline blended with ethanol and biofuel as a fuel which are cleaner than petrol-gasoline and petrol-diesel. Thus, the vehicle consumed these fuels will have a higher strength of engine block for resisting the acidity with higher energy efficiency. In the same way, the electric vehicles are selected for reducing the CO_2_ emissions originated by fossil fuel combustion. In addition, these electric vehicles have higher efficiency than the internal combustion engines, thus, the electricity consumption would be higher in the countermeasure scenario. Similarly, the lesser the CI is, the fewer CO_2_ is generated. While EI has decreased, the CI also reduces. Due to the fact that efficient technologies consume less fuel and also the promotion of ethanol and biofuel, therefore, the CO_2_ emission will be less. Likewise, ES, ECAP and CECap are the indicators for considering the energy used per one person and emitted the CO_2_ emission whist adopting the efficient technologies with ethanol, biofuel and electricity.

For proposed CO_2_ emission mitigation, using alternative fuels is one of the strategies to achieve the goal but their emissions are different from conventional fuels, for instance, CH_4_ emission from compressed natural gas (CNG) and N_2_O emissions from ethanol, etc. (USEPA 2012). Therefore, other air pollutions are considered. The air pollutions of the LCT scenario in the transport sector are analyzed in terms of CO_2_, CH_4_, and N_2_O emissions. The CO_2_ emissions are classified into two sources as CO_2_ emissions from fossil fuels and biogenic-CO_2_ from biofuels. The emission factors are adopted from IPCC (2006) and USEPA (2012).

### Marginal abatement cost (MAC)

This study employs Marginal Abatement Cost (MAC) to determine the priority of the proposed mitigation actions corresponding to passenger and freight transports. As a standard tool for evaluation of economic impacts on CO_2_ mitigation, the MAC curve provides a comparison between total costs of expected CO_2_ countermeasures which are required for emission reduction (in general, in tonnes of CO_2_), compared to the baseline scenario (in general, in USD per tonne of CO_2_). The MAC curve concept ranks the technologies from the lowest abatement cost to the highest one. Therefore, the curve is used for overall comparisons of the emissions reduction opportunities and cost of different technologies. The mathematical formulation of MAC is expressed as follows:

4 $$MAC_{\text{Mitigation}} = \sum\limits_{t = 1}^{T} {\frac{{C_{t}^{\text{Mitigation}} - C_{t}^{\text{Baseline}} }}{{EM_{t}^{\text{Baseline}} - EM_{t}^{\text{Mitigation}} }}}$$

where *MAC*_Mitigation_ is marginal abatement cost of the mitigation scenario,

*C*_*t*_^Mitigation^ is total cost of mitigation scenario in the year *t*,

*C*_*t*_^Baseline^ is total cost of baseline scenario in the year *t*,

*EM*_*t*_^Mitigation^ is total GHG emission of mitigation scenario in the year *t*, and

*EM*_*t*_^Baseline^ is total GHG emission of baseline scenario in the year *t*.

## Scenario development

In this study, end-use driven optimization for Thailand transport planning was conducted under two comparative scenarios which are the Business-as-usual (BAU) and Low Carbon Transport (LCT) scenarios. In the LCT scenario, proposed mitigation actions and policies are included and results are compared to the BAU scenario.

### BAU scenario

The BAU scenario is expressed as a frozen efficiency baseline where no new technologies are implemented and there is no energy efficiency increase from 2010 up to 2050. Future technology and fuel mixes are assumed to maintain a similar pattern as in 2005. Gasoline blended with 10 % of ethanol (E10) was the most important contributor to Thai passenger vehicles where sedans were the largest consumer, accounting for 50.6 % of total gasoline utilization, followed by motorcycles (40.4 %) (DLT 2010). On the other hand, in freight transport, trucks and pick-ups consumed diesel more than 50 %. Although Ethanol-blended products demonstrated success as a replacement of fossil fuel consumption, pure gasoline was still required by 41.4 % of total fuel consumption of passenger transport. For freight transportation, B5 (Diesel blended with 5 % of biofuel) was also used which resulted in the reduction of diesel requirement by 35.9 %.

The costs used in this study have been obtained from reports and literatures. Energy prices in 2005 were obtained from DEDE (2005). The estimated future energy prices have been determined from an escalation rate of 2.00 % (Promjiraprawat and Limmeechokchai 2012). Table 3 provides price setting in the AIM/Enduse model.

**Table 3:** Price setting in the BAU scenario

Fuel	Price (‘000 USD/toe)			
	2005	2020	2030	2050
Gasoline	1.65	2.22	2.70	4.02
Gasohol with ethanol 10 %	1.35	1.81	2.21	3.28
Gasohol with ethanol 20 %	1.28	1.72	2.10	3.11
Gasohol with ethanol 85 %	0.82	1.10	1.34	2.00
High speed diesel	1.06	1.42	1.73	2.58
High speed diesel with 5 % biofuel	1.01	1.37	1.66	2.47
High speed diesel with 10 % biofuel	0.95	1.28	1.56	2.32
High speed diesel with 20 % biofuel	0.85	1.14	1.39	2.06
Liquefied petroleum gas	0.49	0.66	0.81	1.20
Natural gas for vehicle (NGV)	0.41	0.55	0.67	1.00
Fuel oil	0.74	1.00	1.22	1.81
Jet fuel	1.47	1.98	2.42	3.59
Electricity	1.10	1.48	1.81	2.69

Fuel economy used in the AIM/Enduse model is calculated from the obtained range in Thailand’s energy situation report (DEDE 2010). Table 4 provides the fuel economy for various vehicle types corresponding to the specific fuel type.

**Table 4:** Fuel economy in the BAU scenario

Fuel type	Fuel economy by vehicle type (km/litre)									
	Small sedan	Large sedan	Passenger van	Tuk Tuk	Taxi	Motorcycle	Bus	Others	Pickup	Truck
Gasoline	11.59	10.63	8.88	24.33	12.56	37.65	3.86	11.59	5.99	3.86
E10	11.24	10.30	8.61	23.59	12.18	36.51	3.75	11.24	5.81	3.75
E20	10.88	9.98	8.34	22.85	11.79	35.37	3.63	10.88	5.63	3.63
E85	8.42	7.73	6.46	17.69	9.13	27.38	2.81	8.42	4.36	2.81
HSD	15.23	13.28	10.84	29.88	15.72	–	5.47	15.23	10.25	6.74
B5	14.77	12.88	10.51	28.98	15.25	–	5.31	14.77	9.94	6.54
LPG	10.77	8.92	7.15	22.25	11.39	–	3.36	10.77	5.12	3.27
NGV	8.56	7.25	6.12	16.28	8.97	–	3.63	8.56	4.75	3.51

### CO_2_ Countermeasures in the LCT scenario

The World Business Council for Sustainable Development reported on global personal transport activity towards 2050 and showed that car ownership will keep increasing, especially in developing countries (WBCSD 2004). This will increase GHG emissions from light-duty vehicles. The modal shift concept is widely used as a tool to reduce society’s dependence on automobiles. Private vehicles emit more CO_2_ than public transport such as buses and trains; therefore, policy development to push the change of the lifestyle and behavior is vital for modal shift concept. In developed countries, public buses and trains have been provided to serve passenger and freight demands for long distance services. Additionally, urban transport systems such as light rail transit (LRT), bus rapid transit (BRT) and buses have been provided to cope with the demand for short-distance travel in urban areas (Fujimoto 2008). In freight transportation, the modal shift concept has been used to reduce energy requirements, GHG emissions, and costs per traveling demands (USD/t-km); for instance, shifting truck cost in US is 0.170 USD/t-km whereas rail cost is 0.014 USD/t-km. The American Association of State Highway and Transportation Official (AASHTO) reported that 10 % of intercity freight transport by truck was shifted to rail for a saving of 2.50 Mt-CO_2_/yr (Nealer et al. 2012). In additional, fuel switching from fossil-based to low GHG content fuels, (e.g., biofuel and electricity), and efficiency improvement as well as introduction of new advanced technologies are beneficial to reduce fossil fuel dependency and GHG emissions. Biofuels have been widely used in the form of blended-fuels such as E10 and B5. An attempt to increase proportion of biofuel blended appears in the energy policy of many countries. Similar to other countries, in the European Union (EU) the transport sector relies on fossil fuels. The biofuels consumption targets have been set by the Biofuels Directive as 5.75 % in 2010 and increased to 12 % by 2020 (EREC 2008). Biomass-based diesel, cellulosic and advanced biofuel have been in the National Renewable Fuel Standard Program of the United States that targeted conventional biofuel consumption as 15 billion gallons by 2015 and added up to 36 billion gallons by 2022 (USEPA 2010). Furthermore, developing countries such as India also proposed an indicative target of 20 % blending of biofuels for both biodiesel and ethanol by 2017 (MNRE 2013). Additionally, in order to support green fuels, e.g., high blending ratios of biofuels and electricity, and hybrid and plug-in vehicles have been developed.

The Office of Transport and Traffic Policy and Planning (OTP) of Thailand proposed the plan for energy and emission reduction in transportation to support Thailand’s NAMAs. The plan has considered strategies for an effective and sustainable transportation system. Development of transportation infrastructure and mass transport links together with rapid transit system are proposed. Transport demand management and a taxation mechanism to encourage high efficiency vehicles are considered. Finally, encouragement of environmental friendly advanced technologies is proposed. Moreover, the AEDP objective is to promote renewable and alternative energy development to account for 25 % of total final energy consumption. According to the above strategy, ethanol, biodiesel, and new fuel for diesel substitution have been targeted to substitute conventional oil by 44 % in 2021 in the transport sector.

Therefore, following the Thai transport strategies, this study proposes the two main scenarios called “smart passenger transport” for passenger transport policy and “effective freight transport” for freight transport policy. “Smart Passenger Transport” action comprises four main countermeasures which are inclusive of transport demand management (TDM), modal shift, advanced technology, and fuel switching in order to develop appropriate energy and GHG mitigation planning for Thai passenger transport. Also, the three countermeasures, TDM, advanced technology and fuel switching, are taken into account for “effective freight transport” action under NAMAs perspectives.

TDM strategies have been employed in different circumstances which are primarily to manage traffic congestion and GHG emissions mitigation (Kahn Ribeiro et al. 2007). This countermeasure is implemented in both passenger and freight transports by using fuel economy standards in new vehicles, old car retirement, speed limits, work time management, and so on (OTP 2012). It is estimated that TDM can decrease travel demand by 5.02 % in both passenger and freight transports.

A Modal shift countermeasure is proposed only in the passenger transport, and considered three transport modes such as (1) shift from buses to electric trains, (2) non-motorized implementation, and (3) promotion of walkable city. However, due to the high investment cost to construct pathways and political problems in Thailand, the modal shift countermeasure is expected to start in 2030 (OTP 2012).

For advanced technology countermeasures, the four new technologies: hybrid-battery, hybrid-plug in, electric car, and electric railway are applied for the passenger transport. On the other hand, only two new technologies: the hybrid-battery and electric trains are implemented in the freight transport. The technological share setting in the BAU scenario is simple as it is assumed as a frozen efficiency scenario, where the existing devices continue to provide energy services in all systems and are set to be 100 %. The assumptions in the LCT scenario employ new and efficient technologies to reduce GHG emissions available in the studied period of 2005–2050. The minimum technological share settings of new technologies of each device under the relevant category by vehicle types required in the passenger transport sector are shown in Table 5. In all vehicle categories the existing devices will be gradually decreased and the new countermeasure technologies will gain prominently in the latter years. However, due to limitation of technology transfer, this study expects that hybrid-battery technology will be widely used after 2020. Hybrid plug-in vehicles and electric train system will be widely used and in operation after 2030 while electric cars will start in 2040.

**Table 5:** Technological shares of CO_2_ countermeasures in the LCT scenario

Technology			Minimum technological share (%)			
			2005	2020	2030	2050
Passenger transport	Small sedan and taxi	Existing	100.00	70.00	40.00	4.00
		Hybrid	0.00	30.00	48.00	20.00
		Plug-in hybrid	0.00	0.00	12.00	38.00
		Electric vehicle	0.00	0.00	0.00	38.00
	Large sedan	Existing	100.00	70.00	40.00	5.00
		Hybrid	0.00	30.00	60.00	95.00
	Passenger van	Existing	100.00	70.00	40.00	43.00
		Hybrid	0.00	30.00	60.00	57.00
	Motorcycle	Existing	100.00	85.00	70.00	50.00
		Electric vehicle	0.00	15.00	30.00	50.00
	Bus	Existing	100.00	100.00	76.00	60.00
		Electric railway	0.00	0.00	24.00	40.00
	Railway	Existing	100.00	100.00	70.00	40.00
		Electric railway	0.00	15.00	30.00	60.00
Freight transport	Pick-up	Existing	100.00	70.00	46.00	43.00
		Hybrid	0.00	30.00	54.00	57.00
	Railway	Existing	100.00	100.00	70.00	40.00
		Electric railway	0.00	0.00	30.00	60.00

In the fuel switching countermeasure, biodiesel (B5, B10, and B20) and gasohol (E10, E20, and E85) are implemented in the passenger transport, but only biodiesel (B5 and B10) is proposed in the freight transport. While B5 and gasohol have been applied in Thailand since 2010, B10 and B20 need to wait for commercial scale development until 2030 for B10 and 2040 for B20. Table 6 gives the fuel share settings of the vehicle categories in which fuel switching is introduced according to the LCT scenario. Fuel switching is introduced in 7 vehicle types namely; small sedan, large sedan, passenger van, tuk–tuk, motorcycle, bus, and other vehicles. The general fuel shifting happens in three ways: (1) shifting from conventional gasoline to gasoline blended with ethanol, (2) shifting from conventional diesel to diesel blended with biofuel, and (3) shifting to increasing electric vehicles. In the case of ethanol blending, three types of blending are available in Thailand, which are E10, E20, and E85. The E10 is gradually being phased out by the year 2050. It will be substituted by E20 and E85. Likewise, biofuel is obtained in three varieties, which are B5, B10, and B20. These B5 blends are utilized in most categories, whereas the B10 and B20 blends are exclusively used in bus and other vehicle categories (see Table 6).

**Table 6:** Technological shares in fuel switching for passenger transport in the LCT scenario

	Technology	Minimum technology share (%)			
		2005	2020	2030	2050
Small and large sedan	Gasoline	25.97	10.52	0.00	0.00
	Gasoline with ethanol 10 %	35.63	16.20	33.18	0.00
	Gasoline with ethanol 20 %	1.11	21.05	38.71	43.24
	Gasoline with ethanol 85 %	0.01	10.52	22.12	18.01
	High speed diesel	13.09	0.00	0.00	0.00
	High speed diesel with 5 % biofuel	8.21	0.00	0.00	0.00
	Liquefied petroleum gas (LPG)	11.06	6.99	4.00	0.50
	Compressed natural gas (CNG)	4.91	3.49	2.00	0.25
	Electricity	0.00	0.00	0.00	38.00
Passenger van	Gasoline	25.97	3.53	0.00	0.00
	Gasoline with ethanol 10 %	35.63	15.88	33.18	0.00
	Gasoline with ethanol 20 %	1.11	7.06	38.71	70.06
	Gasoline with ethanol 85 %	0.01	3.53	22.12	29.19
	High speed diesel	13.09	24.49	0.00	0.00
	High speed diesel with 5 % biofuel	8.21	38.49	0.00	0.00
	Liquefied petroleum gas (LPG)	11.06	2.09	4.00	0.50
	Compressed natural gas (CNG)	4.91	4.89	2.00	0.25
Tuk Tuk	Gasoline	6.89	0.00	0.00	0.00
	Gasoline with ethanol 10 %	9.44	0.00	0.00	0.00
	Gasoline with ethanol 20 %	0.29	0.00	0.00	0.00
	High speed diesel	0.23	0.00	0.00	0.00
	High speed diesel with 5 % biofuel	0.14	0.00	0.00	0.00
	Liquefied petroleum gas (LPG)	62.39	70.00	70.00	70.00
	Compressed natural gas (CNG)	20.62	30.00	30.00	30.00
Taxi	Gasoline	0.85	3.50	0.00	0.00
	Gasoline with ethanol 10 %	0.75	16.00	10.59	0.00
	Gasoline with ethanol 20 %	0.03	7.00	24.71	40.24
	Gasoline with ethanol 85 %	0.00	3.50	14.12	16.76
	High speed diesel	0.20	0.00	0.00	0.00
	High speed diesel with 5 % biofuel	0.12	0.00	0.00	0.00
	Liquefied petroleum gas (LPG)	19.79	14.00	7.99	0.99
	Compressed natural gas (CNG)	77.72	55.00	31.99	3.99
	Electricity	0.00	0.00	0.00	38.00
Motorcycle	Gasoline	40.34	17.50	10.50	0.00
	Gasoline with ethanol 10 %	35.72	30.00	17.50	0.00
	Gasoline with ethanol 20 %	1.76	12.00	17.50	20.00
	Gasoline with ethanol 85 %	0.00	10.50	24.50	30.00
	Electricity	0.00	15.00	30.00	50.00
Bus	Gasoline	1.43	0.00	0.00	0.00
	Gasoline with ethanol 10 %	1.26	0.00	0.00	0.00
	Gasoline with ethanol 20 %	0.06	0.00	0.00	0.00
	High speed diesel	36.56	50.00	21.00	5.00
	High speed diesel with 5 % biofuel	22.95	50.00	49.00	15.00
	High speed diesel with 10 % biofuel	0.00	0.00	6.00	16.00
	High speed diesel with 20 % biofuel	0.00	0.00	0.00	24.00
	Liquefied petroleum gas (LPG)	2.42	0.00	0.00	0.00
	Compressed natural gas (CNG)	34.53	0.00	0.00	0.00
Other passenger vehicle	High speed diesel	61.43	50.00	24.00	5.00
	High speed diesel with 5 % biofuel	38.56	50.00	56.00	15.00
	High speed diesel with 10 % biofuel	0.00	0.00	20.00	32.00
	High speed diesel with 20 % biofuel	0.00	0.00	0.00	48.00

Table 7 gives the fuel share settings of the vehicle categories for freight transport in the LCT scenario in the AIM/Enduse model. Fuel switching is introduced for pick-ups and trucks. The general fuel shifting happens in two ways: (1) shifting from conventional gasoline to gasoline blended with ethanol and (2) shifting from conventional diesel to diesel blended with biofuel. In the case of ethanol blended with gasoline, the E10 will be gradually phased out by the year 2050. It will be replaced by E20 and E85. Likewise, biofuel is obtained in three varieties, which are (1) B5, (2) B10, and (3) B20. These B5 blends are utilized in most categories, whereas the B10 and B20 blends are exclusively used in bus and other vehicle categories. Another significant aspect to be noted in the share allocation in the device categories is that the share of ethanol is much smaller in comparison to the share of biofuel. This is due to the fact that most pick-ups and trucks use diesel in Thailand.

**Table 7:** Technological shares of fuel switching for freight transport in the LCT scenario

Technology		Technological shares (Minimum)			
		2005	2020	2030	2050
Pick-up	Gasoline	1.80	0.00	0.00	0.00
	Gasohol with ethanol 10 %	2.48	3.82	3.82	0.00
	Gasohol with ethanol 20 %	0.07	4.45	4.45	8.05
	Gasohol with ethanol 85 %	0.00	2.54	2.54	3.35
	High speed diesel	57.23	24.95	24.95	5.55
	High speed diesel with 5 % biofuel	35.92	49.91	49.91	19.45
	High speed diesel with 10 % biofuel	0.00	10.32	10.32	25.65
	High speed diesel with 20 % biofuel	0.00	0.00	0.00	37.43
	Liquefied petroleum gas (LPG)	1.43	1.19	1.19	0.14
	Compressed natural gas (CNG)	0.99	2.79	2.79	0.34
Truck	Gasoline	0.03	0.00	0.00	0.00
	Gasohol with ethanol 10 %	0.03	0.00	0.00	0.00
	High speed diesel	55.57	50.00	16.00	4.00
	High speed diesel with 5 % biofuel	34.88	50.00	64.00	16.00
	High speed diesel with 10 % biofuel	0.00	0.00	20.00	32.00
	High speed diesel with 20 % biofuel	0.00	0.00	0.00	48.00
	Liquefied petroleum gas (LPG)	0.30	0.00	0.00	0.00
	Compressed natural gas (CNG)	9.12	0.00	0.00	0.00

## Results

### Passenger transport

**Fig. 5:**
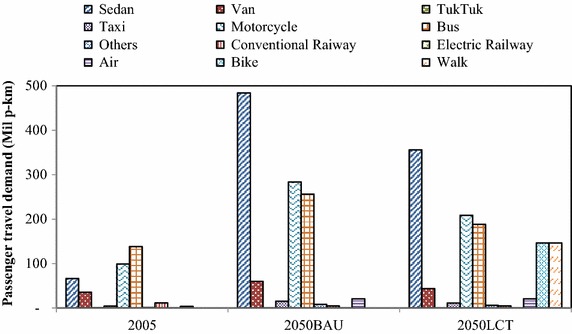
Passenger transport demand

**Fig. 6:**
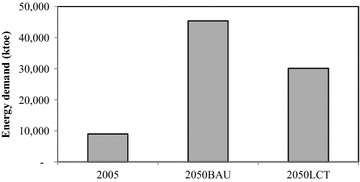
Energy demand in passenger transport

**Fig. 7:**
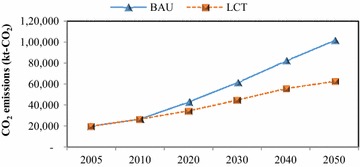
CO_2_ emissions in passenger transport

In the passenger transport sector, implementation of TDM, modal shift, fuel switching, and advanced technologies has been proposed to reduce GHG emissions; this initiative is called the “Smart Passenger Transport” action. In 2050, sedans will have the largest share of passenger travel demand, followed by motorcycles and buses; however, due to the promotion of modal shift, the passenger travel demand for bicycles and walking increases by about 0.43 % in the 2050LCT scenario as shown in Fig. 5. The energy demand of passenger transport will be 45,383 ktoe in the 2050BAU, and reduced to 30,122 ktoe in the 2050LCT, an energy reduction of 33.63 % (see Fig. 6). In 2050LCT, all countermeasures for the “Smart Passenger Transport” action will help to reduce CO_2_ emissions by about 8203 kt-CO_2_, 16,521 kt-CO_2_, and 38,799 kt-CO_2_, accounting for 19.10, 26.89, and 38.21 % of CO_2_ reduction in 2020, 2030, and 2050, respectively (see Fig. 7).

**Fig. 8:**
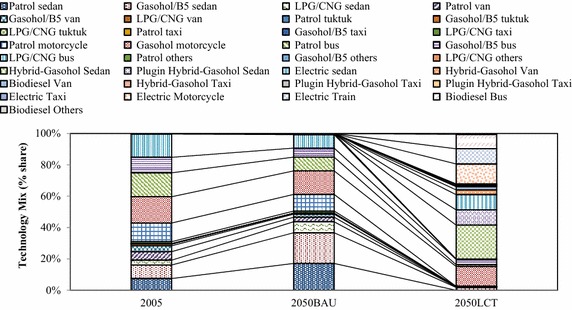
Technological mix in passenger transport in the BAU and LCT scenarios

**Fig. 9:**
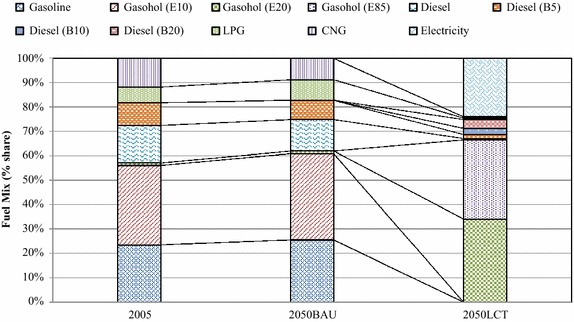
Fuel mix in passenger transport in the BAU and LCT scenarios

In the “Smart Passenger Transport” action, the number of conventional sedans will decrease drastically after 2030 due to technological substitution of hybrid and electric vehicles. This study focuses on land transport. Therefore, low carbon countermeasures in the air and water transports were not taken into consideration. The variety of energy uses are represented in terms of technological mix which are categorized by vehicle types, e.g., conventional vehicles, hybrid vehicles, plug-in hybrid vehicles, etc. The technological mix in Fig. 8 shows the domination of biofuels in the 2050BAU. Hybrid sedans, electric motorcycles, plug-in hybrids, and electric trains will share about 21.80, 12.70, 9.80, and 9.70 % in the 2050LCT scenario. By adoption of advanced technologies for passenger transport in the early period, shares of hybrid vehicles and electric trains will increase drastically and will play an important role in the low-carbon Thailand in the 2050LCT. In the fuel mix, electricity and ethanol will have the largest share in the 2050LCT (Fig. 9). Shares of conventional gasoline, diesel, LPG and CNG are reduced in the 2050LCT.

**Fig. 10:**
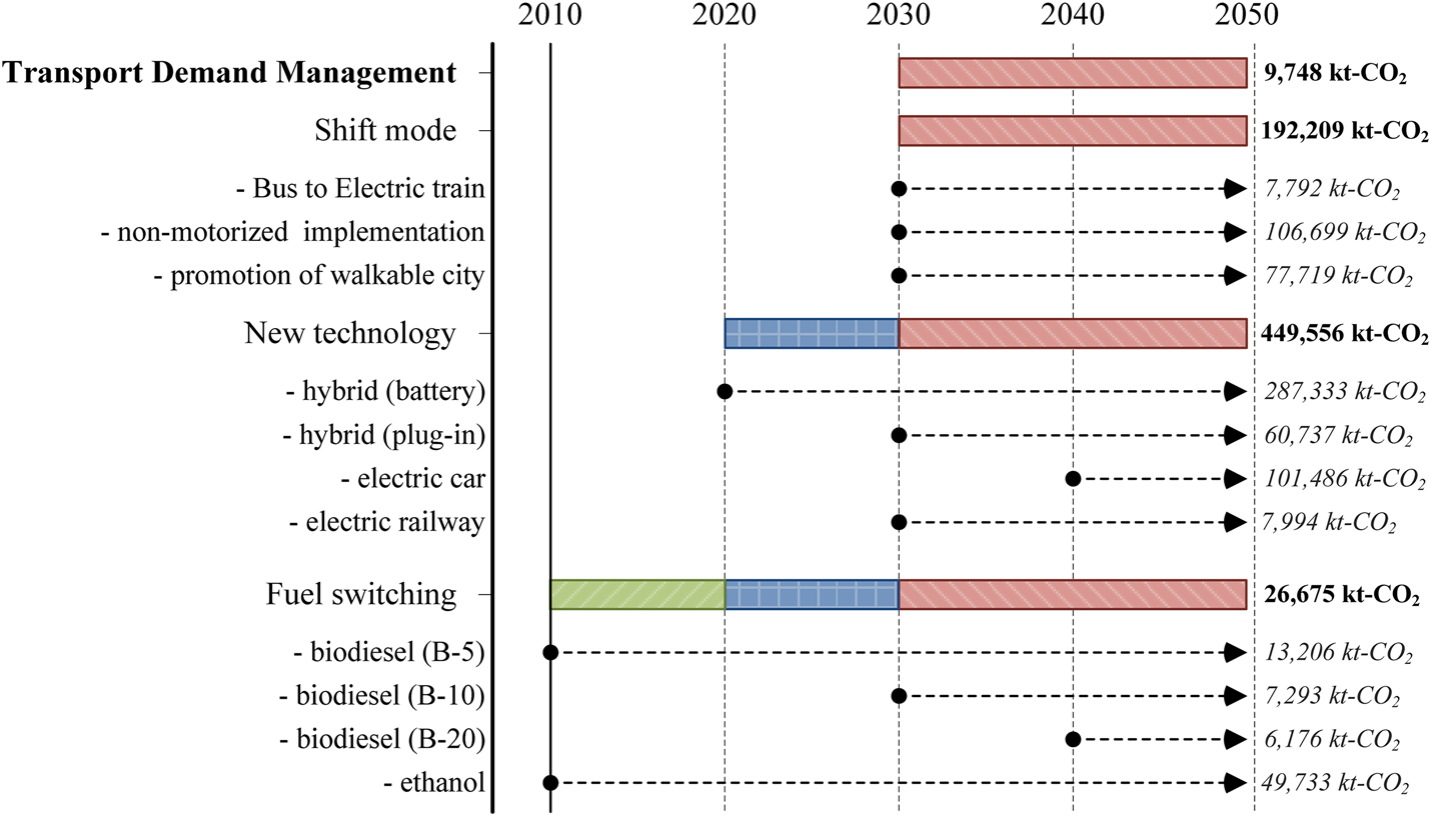
Plans of “Smart passenger transport” action

In the “Smart Passenger Transport” action, GHG countermeasures of biofuels, fuel switching, and hybrid vehicles could be implemented in both short and medium-term periods. Some advanced technologies and countermeasures, such as electric vehicles and biodiesel B20, need to wait for commercial scale development until 2040 (see Fig. 10), while bio-diesel B5 and ethanol have been implemented since 2010. Widespread use of hybrid vehicles in Thailand will happen after 2020. Commercial use of plug-in hybrids will happen after 2030. However, if there is a climate policy to accelerate the commercial uses of such technologies and biofuels at the early stage, GHG reduction levels in 2050 will be higher.

### Freight transport

**Fig. 11:**
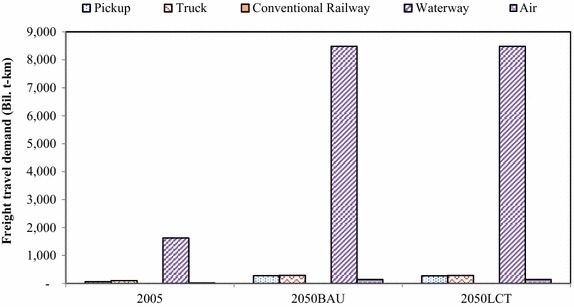
Freight transport demand

**Fig. 12:**
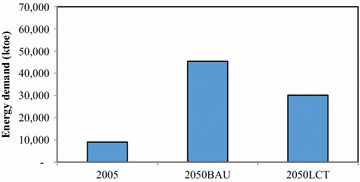
Energy demand in freight transport

**Fig. 13:**
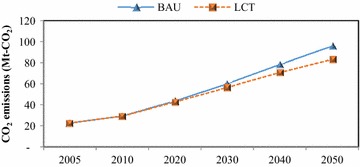
CO_2_ emissions in freight transport

In the freight transport, the “Effective Freight Transport” action is to promote advanced technologies as well as alternative energy for freight transport. In this study, in the freight transport, waterway and air transports will not be taken into LCT analysis due to limitations of available information, even though the waterway is a major freight transportation channel (Fig. 11). Therefore, only countermeasures in the land transport are considered. In the 2050BAU, energy demand of freight transport will increase from 9762 ktoe in 2005 to 40,596 ktoe, or 4.2 times higher than in 2005. In the 2050LCT, all countermeasures of “Effective Freight Transport” action will help reducing energy demand by 1947 ktoe, and resulting in GHG mitigation of 987 kt-CO_2_ (2.26 %), 3502 kt-CO_2_ (5.83 %), and 12,652 kt-CO_2_ (13.2 %) (Figs. 12, 13).

**Fig. 14:**
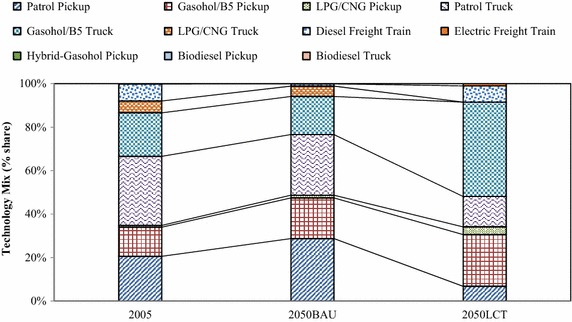
Technological mix in freight transport in the BAU and LCT scenarios

In the “Effective Freight Transport” action, GHG countermeasures in the 2050LCT scenario are focused on land transport while air and water transports are kept the same as in the 2050BAU scenario. The hybrid and bio-diesel (B20) countermeasures will contribute to large shares in the technological mix while conventional technologies will be phased out in 2050 (Fig. 14).

**Fig. 15:**
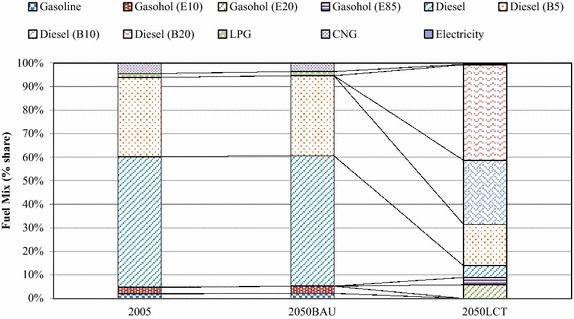
Fuel mix in freight transport in the BAU and LCT scenarios

The fuel mix in Fig. 15 shows that biodiesel will be the main fuel type in the 2050LCT scenario. In the fuel mix, shares of LPG, CNG and gasoline in the “Effective Freight Transport” action will diminish after 2030, while advanced technologies, mass transit system and biodiesel will play an important role in GHG mitigation after 2030.

**Fig. 16:**
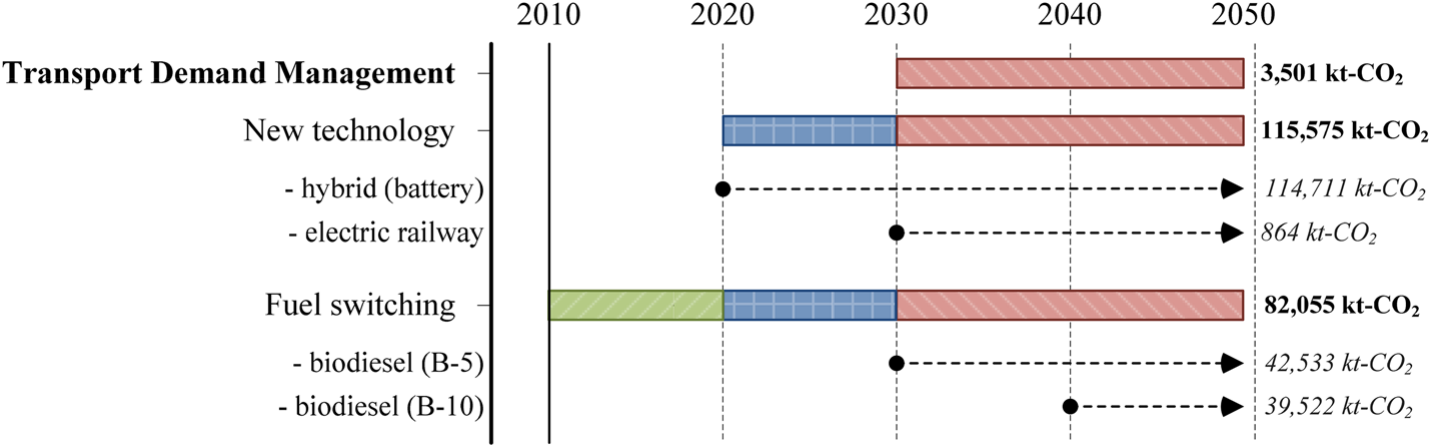
Plans of “Effective freight transport” action

Figure 16 shows the timelines to implement such countermeasures. The most effective GHG countermeasures in the “Effective Freight Transport” action will be biodiesel in the short and medium terms and hybrid diesel engines in the long term.

## Economic and other GHG emission evaluation

### Energy security analysis

**Fig. 17:**
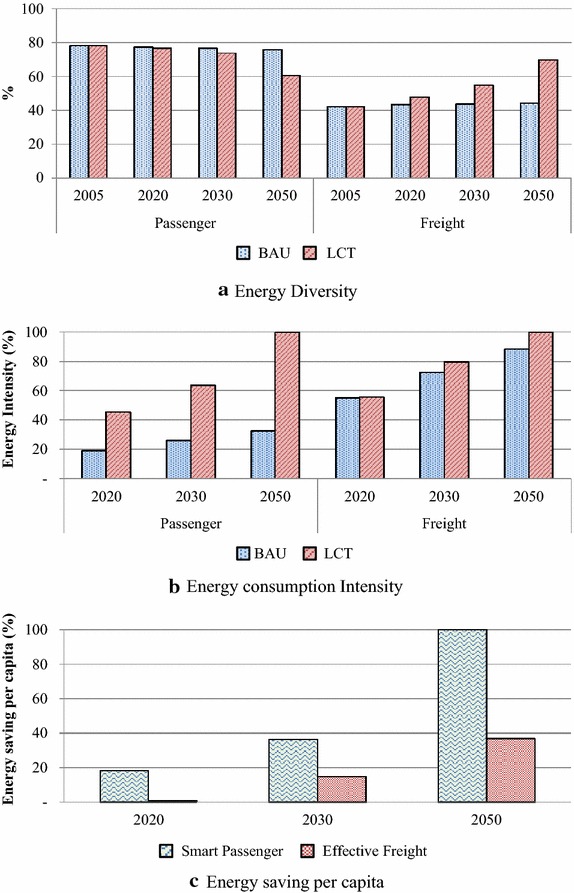
Energy security indicators

The results of energy security of the BAU and LCT scenario are illustrated in Fig. 17. The diversification of energy demand, the ED indicators, given in Fig. 17a, it is almost constant in the BAU scenario for both passenger and freight transports. In the LCT scenario, results show that diversity of energy in the smart passenger plan is slightly decreased since gasoline octane 91 will be phased out and demand for biofuel and electricity will be significantly increased in order to mitigate emissions. On the contrary, the energy diversity of the effective freight action increases due to high speed diesel (HSD) demand, which is dominant in the BAU scenario, and replaced by other fuels such as biodiesel and ethanol.

**Fig. 18:**
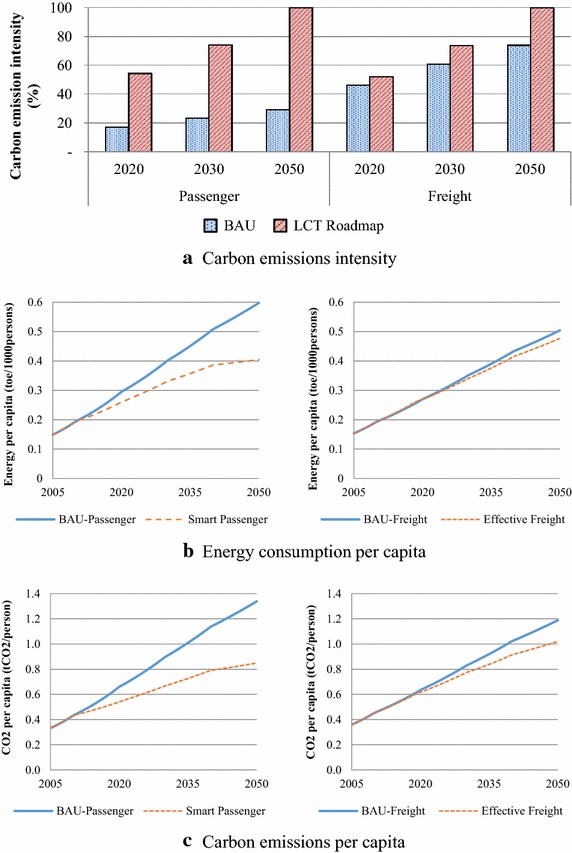
Energy security indicators

The EI and CI indicators are significantly improved in the smart passenger action when compared to the BAU whereas 10–25 % improvement happens in the effective freight transport plan. The smart passenger transport will benefit from the high potential of countermeasures, which include fuel switching to biofuel in the short and medium term, as well as advanced technology, hybrid and electric vehicles in the medium and long term. Moreover, TDM and modal shift measures will play an important role in the long term. In freight transport, the effective freight transport action will be implemented by biodiesel substitution in the short to medium term while hybrid vehicles and TDM action will be implemented in the long term. Its energy demand and GHG emissions mitigation potentials are lower than that of the smart passenger actions. Results from modal shift and advanced technologies in smart passenger transport show effective energy saving which is represented by the ES indicator (see Fig. 17c).

Results of the ECap and CEcap indicators are consistent with other indicators and given in Fig. 18b, c. The LCT scenario consumes less energy and emits less CO_2_. In the smart passenger transport, the ECap and CECap indicators are significantly decreased whereas the effective freight transport indicators are similar to the BAU scenario. The reductions of CO_2_ emissions in the CECap indicator for both smart passenger and effective freight transports are higher than the reduction in energy demand in the ECap indicators due to the effect of fuel switching to biofuel, which has lower heating content (DEDE 2010; Bell et al. 2011). Thus, more biofuels are required for the same demand.

### Other GHG emission analysis

Besides CO_2_ emission, N_2_O and CH_4_ are also emitted during the combustion of fossil fuel and biofuel in vehicles. The amount of N_2_O and CH_4_ emissions comprises a relatively small proportion of overall transportation related GHG emissions. This analysis is limited to other GHGs, excluding other air pollutions. In the BAU scenario, N_2_O emission mostly comes from fossil fuels combustion in the passenger and freight transports. It increases from 0.35 and 0.11 Mt-CO_2_eq in the base year to 1.46 and 0.38 Mt-CO_2_eq in 2050, accounting for 3.21 and 2.79 % average annual growth rates, respectively. Similarly, CH_4_ emission in the BAU scenario is emitted from fossil sources with average increasing rates of 2.83 and 2.55 % per year for the passenger and freight vehicles. The increase of these emissions mainly comes from LPG and CNG, which are continuously consumed in the BAU scenario.

In the LCT scenario, the energy demand is decreased and switched from fossil fuels to biofuels as well as electricity. In the passenger transport, the main fuels are gasoline and gasohol in the base year whereas biofuels will be the major fuel consumed in the long run. CH_4_ and N_2_O emissions from biofuel combustion are higher than that of fossil fuels. Although the contribution of these emissions are minor when compared to CO_2_ emission, the drastic increase of biofuels utilization in passenger transport in the LCT scenario results in the increase of N_2_O emissions, which show an average annual growth rate of 4.40 %. In the freight transport, diesel and CNG are mainly used in the base year and they will be switched to biofuels, but not significantly. The average increasing N_2_O emission from effective freight actions is 2.86 % per year during 2005–2050. The reduction of LPG and CNG in the LCT scenario strongly affects CH_4_ emissions. CH_4_ emission from smart passenger actions increases from 3.26 kt-CO_2_eq in 2005 to 4.59 kt-CO_2_eq whereas the reduction on LPG and CNG in effective freight actions results in the decrease of CH_4_ emissions from 1.23 kt-CO_2_eq in 2005 to 1.00 kt-CO_2_eq in 2050.

CO_2_ emissions in the transport sector are classified as CO_2_ emissions from fossil fuel and biogenic-CO_2_ emission which is generated during biofuel combustion. As biofuels are promoted to be used in the LCT scenario, the biogenic-CO_2_ emission from this scenario is higher than the BAU scenario (about 4 and 3 times compared to the BAU for passenger and freight transport, respectively). But, overall CO_2_ emissions in the LCT scenario is lower than in the BAU scenario due to the counter measures of energy efficiency via advanced technology vehicle utilization as well as switching to low emission fuels. The cumulative N_2_O, CH_4_ and CO_2_ emissions in the BAU and LCT scenarios are shown in Table 8.

**Table 8:** Cumulative N_2_O, CH_4_ and CO_2_ emissions in the BAU and LCT scenarios

Unit:Mt-CO_2_eq							
		Fossil fuels			Biofuels		
		N_2_O	CH_4_	CO_2_	N_2_O	CH_4_	CO_2_
BAU scenario	Passenger						
	Private car	18.56	0.14	1,240.84	3.81	0.01	48.40
	Taxi & Tuk tuk	2.99	0.06	34.03	0.01	0.00	0.05
	Bus	4.87	0.08	120.00	0.09	0.00	1.99
	Motorcycle	4.36	0.04	314.37	5.38	0.01	17.44
	Others	0.20	0.00	30.14	0.00	0.00	0.70
	Freight						
	Pickup	5.23	0.03	1770.15	0.28	0.00	43.27
	Truck	5.24	0.08	363.68	0.00	0.00	7.58
LCT scenario	Passenger						
	Private car	8.21	0.07	606.82	26.67	0.04	229.52
	Taxi & Tuk tuk	1.26	0.02	15.97	0.26	0.00	1.82
	Bus	0.98	0.01	80.39	0.00	0.00	4.28
	Motorcycle	1.59	0.01	125.33	28.67	0.04	120.47
	Others	0.01	0.00	23.67	0.00	0.00	1.47
	Freight						
	Pickup	5.97	0.07	1608.66	3.40	0.01	168.62
	Truck	2.43	0.03	327.25	0.00	0.00	37.89

### Marginal abatement cost (MAC)

**Fig. 19:**
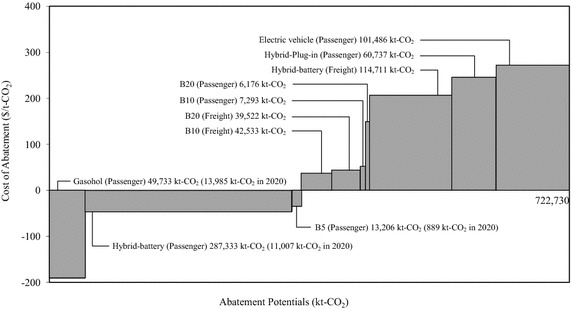
Abatement cost curve of the road transport sector

Most transport vehicles have lower initial investment cost than energy cost. Hence, a vehicle with fuel savings implies not only CO_2_ emission reduction but also cost saving as well as a negative MAC. As shown in Fig. 19, ethanol-blended products, especially E85, would be the most cost-effective mitigation option of fuel switching in the future passenger transport. The first generation biofuel (B5) would also be a competitive fuel. Hybrid-battery vehicles in the passenger transport offer various remarkable benefits in terms of cost savings and CO_2_ mitigation. They have mitigation potential of about 287.33 Mt-CO_2_ with low abatement cost of about −45.90 $/t-CO_2_, which shows the cost-effectiveness of this technology. Nevertheless, advanced technologies with electricity consumption such as plug-in hybrids and electric vehicles with high MACs would require high incentives in order to sustain expensive imported vehicles and rapid growth of electricity demand.

For freight transport, advanced technology and biofuel switching would still be an expensive option according to evidence of a high positive MAC. Although hybrid pick-ups would be the greatest mitigation alternative, practical introduction of advanced technology would pose a budgetary burden. Results show that the hybrid-battery, hybrid-plug in, and electric car will mitigate CO_2_ by about 276.93 Mt-CO_2_. However, even if hybrid-battery technology in the freight transport and electric vehicles in the passenger transport have high potential to mitigate CO_2_, they also have high abatement costs of about 205.90 $/t-CO_2_ and 271.70 $/t-CO_2_, respectively.

Moreover, biofuel switching is a more cost-effective approach to mitigate CO_2_ emission, despite resulting in a positive MAC. Introduction of the third generation biofuel (B20) would be well-matched to the second generation biofuel (B10) in terms of comparative MAC. Both B10 and B20 will reduce CO_2_ emissions by about 95.52 Mt-CO_2_. However, because more than about 80 % of biofuels are used in freight vehicles (DLT 2010), the abatement costs of biofuel technologies in these vehicles are lower than in the passenger vehicles.

Under Thailand’s proposed transport NAMAs, the criteria of abatement cost in terms of NAMAs is defined at 10$/t-CO_2_ (TU-RAC 2012), which means that if the abatement costs of countermeasures are lower than 10$/t-CO_2_, they will be categorized as DSMA. On the other hand, abatement costs which are higher than 10$/t-CO_2_ will be classified as ISMA. Therefore, results indicate that three countermeasures in the passenger transport such as implementation of gasohol, hybrid-battery, and B5, which could reduce CO_2_ emissions by 25,881 kt-CO_2_ in 2020 and accounted for about 54.04, 42.53, and 3.43 % of overall CO_2_ mitigation potential in gasohol, hybrid-battery, and B5, respectively, would be proposed to be DSMA. Even if the hybrid-battery technology has not been proposed before 2020 in the mitigation plan, results show that this technology has high potential to reduce CO_2_ emissions with cost effectiveness. Therefore, the government should immediately plan to promote the hybrid-battery technology.

In contrast, advanced technologies with high MACs such as B10 and B20 biodiesel, hybrid technology, and electric vehicle would be implemented under the ISMA, which require both financial support and technology transfer from developed countries. However, in this study, the advanced technologies are proposed to be implemented after 2020. For the above reasons, therefore, Thailand’s NAMAs should start on the implementation of effective countermeasures such as gasohol, hybrid-battery, and B5. On the other hand, in the freight transport, not only B10 but also B20 have high GHG mitigation potential; therefore, the Thai government should prepare plans and strategies in order to apply for international support, and encourage early implementation with higher GHG emissions reduction.

## Conclusions and recommendations

This study proposed four mitigation actions in the passenger transport and three mitigation actions in the freight transport in order to investigate the energy savings as well as CO_2_ mitigation. Moreover, results have been applied in the analysis of (1) energy security in terms of six indicators which are energy diversity, energy intensity, carbon intensity, energy consumption per capita, CO_2_ per capita, and energy savings per capita, (2) other air pollutions evaluation including N_2_O, CH_4_, and biogenic CO_2_, and (3) economic aspect in terms of MAC to determine the cost of CO_2_ reduction by technology. In the BAU scenario, results indicate that energy demand and CO_2_ emissions in the transport sector will increase by 67,206 ktoe and 154,263 kt-CO_2_ in 2050, respectively when compared to 2005 level. In the “Smart Passenger Transport” action, energy demand of passenger transport will be 45,383 ktoe in the 2050BAU scenario, and reduced to 30,122 ktoe in the 2050LCT scenario, accounting for 33.63 % of total energy reduction. CO_2_ emissions in the 2050LCT scenario will be reduced by 38.21 % when compared to the 2050BAU scenario level, whereas both the new technology and the transportation shift mode are the efficient countermeasures to reduce CO_2_ emissions in this action. In the “Effective Freight Transport” action, all countermeasures will help to reduce energy demand by 1947 ktoe, resulting in CO_2_ mitigation of 12,652 kt-CO_2_, or about 13.2 %. The most effective mitigation countermeasures in this action will be the implementation of hybrid-battery and promotion of biodiesel which will reduce CO_2_ emissions by about 97.8 % of overall CO_2_ mitigation from this action.

All countermeasures in the LCT scenarios also improve energy security of GHG mitigation contexts. In short and medium-term, fuels switching and advanced technologies of vehicles play an important role in reducing energy demand and GHG emissions. TDM and modal shift actions implemented after 2030 are important to long term GHG mitigation. Therefore, in the NAMA’s target year 2020, the improvements on the EI and CI in smart passenger actions are 26.46 and 37.15 %, respectively, while their effective freight actions are improved to 0.59 and 5.97 %, respectively. These indicators are dramatically enhanced in 2030 and 2050. In 2050, the EI and CI for smart passenger actions are improved to 67.52 and 70.74 % while effective freight actions are 11.62 and 26.08 %, when compared to the 2050BAU, respectively. The countermeasures also affect the diversity of energy. The ED in the smart passenger action is slightly decreased since gasoline octane 91 will be phased out and because of increasing demand of biofuel and electricity whereas the ED of effective freight increases due to biodiesel and ethanol consumption. In addition, results on the ECap and CEcap indicators are consistent with other indicators as the LCT scenario consumes less energy and emits less CO_2_ emission, especially in the long-term period. While fossil fuels will be replaced by biofuels which generate more CH_4_ and N_2_O emissions, the TDM and modal shift actions, including low energy consumption in advanced technology vehicles, gives less cumulative emissions during 2005–2050.

Finally, this study investigates cost effectiveness of GHG emission countermeasures by using MACs. Results show that the hybrid-battery and electric vehicles will be the important technologies to reduce CO_2_ emissions which will mitigate CO_2_ by about 503.53 Mt-CO_2_ in the 2050LCT scenario. However, even if hybrid-battery technology in the freight transport and electric cars in the passenger transport have high potential to mitigate CO_2_, they have high abatement cost, about 205.90 $/t-CO_2_ and 271.70 $/t-CO_2_, respectively. On the other hand, the hybrid-battery in the passenger transport has not only mitigate 287.33 Mt-CO_2_ but also provide lower abatement cost of -45.90 $/t-CO_2_ which shows the cost-effectiveness of the technology. Additionally, using gasohol and B5 biodiesel can mitigate CO_2_ by about 62.94 Mt-CO_2_ in 2050 with low abatement cost of about −190.50 $/t-CO_2_ and −34.40 $/t-CO_2_. However, under Thailand’s proposed transport NAMAs in 2020, implementation of gasohol, hybrid-battery, and B5 biodiesel would be proposed as DSMAs, where low abatement cost of these technologies could reduce CO_2_ emissions by 25.88 Mt-CO_2_ in 2020. On the other hand, both B10 and B20 have high GHG mitigation potential. Hence, they should be proposed as ISMA to encourage more GHG emission reduction. However, there is a limitation of shadow price availability in Thailand’s transport sector and needed in depth analysis. Therefore, the competitiveness of biofuels and electricity and shadow price availability using in both passenger and freight transport are recommended for further study in Thailand’s transport sector.
